# Ovulation induction regimens are associated with a higher rate of livebirth after frozen single-blastocyst transfer among women with polycystic ovary syndrome

**DOI:** 10.3389/fendo.2022.987813

**Published:** 2022-08-15

**Authors:** Yue Niu, Dingying Zhao, Yuhuan Wang, Lu Suo, Jialin Zou, Daimin Wei

**Affiliations:** ^1^ Center for Reproductive Medicine, Cheeloo College of Medicine, Shandong University, Jinan, China; ^2^ Key Laboratory of Reproductive Endocrinology of Ministry of Education, Shandong University, Jinan, China; ^3^ Medical Integration and Practice Center, Shandong University, Jinan, China

**Keywords:** frozen embryo transfer, hormone replacement therapy, ovulation induction, polycystic ovary syndrome, single-blastocyst transfer

## Abstract

**Background:**

Hormone replacement therapy (HRT) regimen was suggested to be associated with a decreased rate of livebirth and a higher risk of hypertensive disorders of pregnancy (HDP) after frozen cleavage stage embryo transfer in women with polycystic ovary syndrome (PCOS). With the dramatically increased use of elective single embryo transfer, there is great need to explore the impacts of different endometrial preparation regimens on frozen single-blastocyst transfer in women with PCOS.

**Methods:**

In this study, a total of 3941 women who diagnosed with PCOS and underwent single-blastocyst transfer during their first cycles of frozen embryo transfer (FET) between March 2012 and December 2020 were included. We retrospectively compared the pregnancy and neonatal outcomes after frozen single-blastocyst transfer with endometrial preparation by HRT regimen (n = 3540), ovulation induction by human menopausal gonadotropin (hMG) regimen (n = 226), and ovulation induction by letrozole regimen (n = 175).

**Results:**

After adjustment for confounders with multivariable logistic regression, the hMG regimen group [(58.4% vs. 49.6%; adjusted odds ratio (aOR): 1.43; 95% confidence interval (CI): 1.09-1.89)] and letrozole regimen group (58.9% vs. 49.6%; aOR: 1.42; 95% CI: 1.04-1.93) were associated with a higher rate of livebirth (primary outcome), compared with the group with HRT regimen. As to the secondary outcomes, the rate of pregnancy loss in the hMG regimen group (22.8% vs. 30.3%; aOR: 0.69; 95% CI: 0.48-1.00) and letrozole regimen group (16.9% vs. 30.3%; aOR: 0.48; 95% CI: 0.30-0.78) was also lower than that in the HRT regimen group. The pregnancy outcomes between the hMG regimen group and the letrozole regimen group were similar. We did not observe significant difference in the incidences of maternal and neonatal complications among these three groups.

**Conclusion:**

Ovulation induction regimen with letrozole or hMG for endometrial preparation was associated with a higher livebirth rate and a lower pregnancy loss rate in frozen single-blastocyst transfer cycles among women with PCOS.

## Introduction

Polycystic ovary syndrome (PCOS) is the most common endocrine disorder in reproductive age women (1), and is a major cause of anovulatory infertility. In women with PCOS who underwent *in vitro* fertilization (IVF), our multicenter randomized trial demonstrated freeze-only strategy with deferred frozen embryo transfer (FET) increased the rate of livebirth while decreased the risks of ovarian hyperstimulation syndrome (OHSS) and pregnancy loss compared with fresh embryo transfer (2). FET has been a routine treatment for patients with PCOS in many assisted reproductive technology (ART) centers (3). Furthermore, the use of elective single-blastocyst transfer has been increasingly advocated in FET cycles for an increased livebirth rate and a reduced multiple pregnancy rate (4).

Due to the feature of oligoovulation/anovulation, women with PCOS need hormone replacement therapy (HRT) regimen or ovulation induction regimen for endometrial preparation before FET. For years, HRT regimen was recognized as the first line endometrial preparation protocol before FET in women with PCOS for easily planning (5), decreased rate of cancellation (6), and minimized risk of OHSS. However, HRT regimen was associated with an increased risk of hypertensive disorders of pregnancy (HDP) compared with natural ovulatory cycle, possibly due to its lack of corpus luteum (7). Regarding to the rates of pregnancy and livebirth, in ovulatory women, earlier studies suggested similar efficacy between the natural ovulation regimen and the HRT regimen (8–10). Several studies have focused on the pregnancy outcomes of different endometrial preparation regimens in women with ovulatory dysfunction (11–16). However, most of these studies employed cleavage-stage embryo transfer (11, 12, 14–16), and the efficiency of different ovulation induction regimens was rarely explored. It remains unknown whether ovulation induction regimen is superior to HRT regimens in single-blastocyst transfer cycles, and which medicine is better for ovulation induction regimen is yet to be established.

A recent cochrane meta-analysis concluded that for women diagnosed with PCOS who underwent ovulation induction and timed intercourse, letrozole appears to improve live birth and pregnancy rates compared with clomiphene citrate (17). Furthermore, it was demonstrated by another cochrane meta-analysis that in women with clomiphene citrate-resistant PCOS, gonadotrophins resulted in more live births than continued clomiphene citrate (18). The study by Lin et al. showed that the use of letrozole was associated with improved clinical pregnancy outcomes compared with human menopausal gonadotropin (hMG) in ovulatory patients who underwent FET (19). However, the efficiency of letrozole compared with gonadotropin was rarely compared in women diagnosed with PCOS. Several studies revealed that letrozole improved endometrial receptivity as compared with clomiphene in patients with PCOS (20–22). Nonetheless, whether letrozole used for ovulation induction regimen could improve the outcome of frozen single-blastocyst transfer still lacks of evidence and warrants further studies.

In this study, we compared the rates of pregnancy and livebirth as well as the risks of obstetric and neonatal complications among women with PCOS who underwent HRT regimen, hMG ovulation induction regimen, or letrozole ovulation induction regimen for endometrial preparation in frozen single-blastocyst transfer cycles.

## Materials and methods

### Patients

This was a retrospective study. A total of 3941 infertile women with PCOS were included this study at the Center for Reproductive Medicine of Shandong University from March 2012 to December 2020. Eligible women were aged 20–40 years and diagnosed with PCOS, all of them underwent single-blastocyst transfer at their first attempts of IVF with or without intracytoplasmic sperm injection (ICSI), and the included cycles were their first FET cycles. To diagnose PCOS, we used Rotterdam consensus (23) as fulfilling at least two of the three criteria and excluding other causes of hyperandrogenism and ovulation dysfunction: oligoovulation or anovulation; clinical or biochemical hyperandrogenism; and polycystic ovarian morphology on ultrasound, as defined by at least one ovary with ≥12 follicles or volume ≥10 cm^3^. Preimplantation genetic test cycles were excluded from this study. Women with abnormal intrauterine cavity (such as a uterine malformation, adenomyosis, submucous myoma, or intrauterine adhesion), preexisting diabetes and hypertension before pregnancy, or a history of recurrent miscarriage (defined as three or more previous spontaneous pregnancy losses) were also excluded. The study protocol was approved by the Institutional Ethics Committee of the Center for Reproductive Medicine of Shandong University (Ethical Review No.27, 2021).

### Embryo culture, evaluation, and selection or transfer

GnRH antagonist protocol or GnRH agonist protocol was used for ovarian stimulation as previously reported (2, 24, 25). Once oocytes retrieval was done after ovarian stimulation, conventional IVF or ICSI was performed approximately 4 to 6 hours later. After 16–18 hours, fertilization was confirmed by the presence of two pronuclei and two polar bodies. On day 3 of embryo culture, embryos were graded by morphological criteria on the basis of the number and size of blastomere and the percentage of fragmentation (26), and the culture medium was changed. The quality of single-blastocyst was assessed according to the Gardner morphological criteria (27). Blastocysts were vitrified on day 5, day 6 or day 7 according to embryo development. For thawing, embryos were transfer into dilution solution in a sequential manner. Blastocyst with score of 4BC or better was transferred. All patients received single-blastocyst transfer.

### Endometrial preparation before embryo transfer

The selection of the regimens for endometrial preparation was based on physicians’ experience and patients’ preferences.

In the hormone replacement therapy cycle regimen, oral oestradiol valerate (Progynova, Delpharm Lille, Lys-Lez-Lannoy, France) at a dose of 4–6 mg daily was started on cycle day 1–3. Vaginal progesterone gel (Crinone, Merck Serono, Watford, UK) 90 mg per day and oral dydrogesterone (Duphaston, Abbott, OLST, Netherlands) 10 mg twice daily were added when the endometrial thickness reached ≥8 mm. And then the transfer was carried out 5 days after progesterone initiation. If pregnancy was achieved, oral oestradiol valerate was continued until 8 weeks’ gestation, and vaginal progesterone gel and oral dydrogesterone were continued until 12 weeks’ gestation.

In the group underwent hMG stimulated, 75 IU/day of hMG (Le Baode, Livzon, Zhuhai, China) was injected from cycle day 3 for 5 days. Ultrasound monitoring started on days 8 of the menstrual cycle, and the dose of hMG was adjusted according to follicle development. Based on the diameter of the dominant follicle was ≥17 mm or the occurrence of an LH surge, human chorionic gonadotrophin (hCG, Le Baode, Livzon, Zhuhai, China) was administered for triggering ovulation. Oral dydrogesterone 10 mg twice daily was administered for luteal phase support after ovulation, and the transfer of embryos was performed on the 5th days after ovulation. If pregnancy was achieved after frozen blastocyst transfer, luteal phase support was continued until 11 weeks’ gestation.

For the letrozole stimulated group, the patients orally took 2.5–5 mg of letrozole per day (Laiquzuo, Heng-Rui, Jiangshu, China) for 5–7days, from day 3 of menstrual cycle. Ultrasound monitoring started on days 8 of the menstrual cycle. If the dominant follicle reached a diameter of ≥10 mm on cycle day 10, transvaginal ultrasound was repeated every 2 days and no other drug was added until ovulation triggering. If a dominant follicle was <10 mm on day 10, a daily dosage of 75–150 IU hMG was supplemented to stimulate follicle growth. The other procedure was the same as in the hMG group.

### Study outcomes

The primary outcome was livebirth after the frozen single-blastocyst transfer. Secondary outcomes were endometrial thickness, biochemical pregnancy, clinical pregnancy, ongoing pregnancy, pregnancy loss, and birth weight. Safety outcomes included ectopic pregnancy and other obstetrical and perinatal complications [i.e., preterm delivery, gestational diabetes mellitus (GDM), HDP, small-for-gestation-age (SGA) neonates, large-for-gestation-age (LGA) neonates, congenital anomalies]. Livebirth was defined as the delivery of any viable neonate who was 28 weeks of gestation or older. Biochemical pregnancy was defined as a serum hCG level >10 IU/L at 12–14 days after FET. Clinical pregnancy was defined as the presence of at least one gestational sac in the uterine cavity on ultrasound at 5 weeks after FET. Ongoing pregnancy was defined as available pregnancy after 12 weeks’ gestation. Miscarriage was defined as pregnancy loss before the 28th gestational week. Preterm birth was defined as delivery before 37 weeks of gestation. GDM was defined as one or more of the 75 g OGTT glucose level indexes above the following cut-off values screened between 24–28 weeks of gestation after excluding pregestational diabetes mellitus:fasting plasma glucose ≥5.1mmol/l; 1 h plasma glucose ≥10.0 mmol/l; 2 h plasma glucose ≥8.5 mmol/l (28). HDP was defined as sustained blood pressure ≥140/90 mmHg after 20 weeks, including gestational hypertension, pre-eclampsia, eclampsia and without a prior history of hypertension (29). SGA was defined as birthweight lower than the 10th percentile of referential birthweight. LGA was defined as birthweight higher than the 90th percentile of referential birthweight. Low birth weight was defined as birth weight smaller than 2500 g, and macrosomia was defined as birth weight greater than 4000 g.

### Statistical analysis

For continuous variables, the normality was tested by the graphical use of histograms, Q–Q plots and the Shapiro–Wilk test. If data are normally distributed, then they were described as mean ± standard deviation (SD); otherwise, they were presented as median (25th percentile to 75th percentile). Categorical variables were expressed as number of cases (n) and percentage of occurrence (%). Continuous data were compared with the use of one way ANOVA test or Kruskal-Wallis test. Categorical variables were assessed by χ² analysis and Fisher’s exact test. Pairwise comparisons among groups were performed with the Bonferroni method to modify the significance levels. Multivariable logistic regression analysis was used to evaluate the possible relationship between the type of endometrial preparation and outcomes after adjusting for confounding factors, including age, body mass index, duration of infertility, gravidity, year of treatment, and number of oocytes retrieved. All statistical analyses were performed by Statistical Package for Social Sciences (SPSS) version 26.0. A *P* value <0.05 was considered to be statistically significant.

## Results

### Baseline characteristics

As is illustrated in **Table 1**, the three groups exhibited similar baseline characteristics, including age, body mass index, gravidity, parity, infertility causes, fertilization method, donor sperm, ovarian stimulation protocols, days of ovarian stimulation, total gonadotropin dose, and number of oocytes retrieved. The HRT group had a longer duration of infertility compared to the hMG group [4.0 (2.5-5.5) vs. 3.0 (2.0-5.0), *P*=0.021]. There was a significant difference in the year of treatment among the HRT regimen, hMG regimen and letrozole regimen groups (*P*=0.273 for HRT regimen group vs. hMG regimen group, *P*<0.001 for HRT regimen group vs. letrozole regimen group, *P*=0.057 for hMG regimen group vs. letrozole regimen group). In addition, the letrozole regimen achieved thicker endometrium [10.0 (8.5-11.0) vs. 9.0 (8.5-10.0), *P*=0.001] compared with the HRT group.

**Table 1:** Basic characteristics of the participants at cycle level by the different protocols.

Characteristic	HRT regimen (n = 3540)	hMG regimen (n = 226)	Letrozole regimen (n = 175)	*P* Value
Age (years)	29.0 (27.0-31.0)	28.0 (26.0-31.0)	28.0 (26.0-30.0)	0.155
Body mass index (kg/m²)	24.6 (21.9-27.4)	24.2 (21.6-27.2)	24.6 (22.4-27.3)	0.419
Duration of infertility (years)	4.0 (2.5-5.5)^a^	3.0 (2.0-5.0)	3.5 (2.0-5.0)	0.013
Gravidity (%)				0.110
0	2380 (67.2%)	136 (60.2%)	110 (62.9%)	
1	780 (22.0%)	56 (24.8%)	46 (26.3%)	
≥2	380 (10.7%)	34 (15.0%)	19 (10.9%)	
Parity (%)				0.389
0	3232 (91.3%)	201 (88.9%)	157 (89.7%)	
≥1	308 (8.7%)	25 (11.1%)	18 (10.3%)	
Infertility causes (%)				0.373
PCOS only	265 (7.5%)	13 (5.8%)	15 (8.6%)	
PCOS + tubal factor	2112 (59.7%)	145 (64.2%)	102 (58.3%)	
PCOS + male factor	265 (7.5%)	15 (6.6%)	17 (9.7%)	
PCOS + other factors	75 (2.1%)	9 (4.0%)	5 (2.9%)	
PCOS + mixed factors	823 (23.2%)	44 (19.5%)	36 (20.6%)	
Fertilization method (%)				0.483
IVF	2824 (79.8%)	185 (81.9%)	140 (80.0%)	
ICSI	572 (16.2%)	28 (12.4%)	28 (16.0%)	
Half IVF + half ICSI	144 (4.1%)	13 (5.8%)	7 (4.0%)	
Donor sperm (%)	175 (4.9%)	12 (5.3%)	5 (2.9%)	0.435
Year of treatment (%)				<0.001
2012-2014	499 (93.3%)^b^	29 (5.4%)	7 (1.3%)	
2015-2016	1122 (87.0%)^a,b^	90 (7.0%)	77 (6.0%)	
2017-2018	954 (90.3%)	53 (5.0%)	50 (4.7%)	
2019-2020	965 (91.0%)	54 (5.1%)	41 (3.9%)	
Ovarian stimulation protocols (%)				0.663
Long agonist protocol	1807 (51.0%)	108 (47.8%)	94 (53.7%)	
Short agonist protocol	86 (2.4%)	10 (4.4%)	3 (1.7%)	
GnRH antagonist protocol	1610 (45.5%)	106 (46.9%)	76 (43.4%)	
Other protocols	37 (1.0%)	2 (0.9%)	2 (1.1%)	
Days of ovarian stimulation (days)	11.0 (9.0-13.0)	11.0 (9.0-13.0)	11.0 (9.0-13.0)	0.755
Total gonadotropin dose (IU)	1525.0 (1200.0-2250.0)	1500.0 (1200.0-2250.0)	1481.3 (1125.0-2325.0)	0.769
Number of oocytes retrieved (n)	17.0 (12.0-21.0)	16.0 (12.0-20.0)	17.0 (13.0-22.0)	0.134
Endometrium thickness (mm)	9.0 (8.5-10.0)^b^	9.0 (8.0-10.0)	10.0 (8.5-11.0)	0.001

HRT, hormone replacement therapy; hMG, human menopausal gonadotropin; PCOS, polycystic ovary syndrome; IVF, *in vitro* fertilization; ICSI, intracytoplasmic sperm injection. ^a^ There were significant differences between the HRT group and the hMG group. ^b^ There were significant differences between the HRT group and the Letrozole group.

### Reproductive outcomes

The comparison of the reproductive and neonatal outcomes between the three groups is presented in **Table 2**. The rates of livebirth (58.4% vs. 49.6%, *P*=0.030) and ongoing pregnancy (60.2% vs. 51.5%, *P*=0.033) were higher in the hMG regimen group compared with the HRT regimen group. The rate of livebirth was of borderline statistical difference between the letrozole regimen group and the HRT regimen group (58.9% vs. 49.6%, *P*=0.051). The letrozole regimen group also had a lower rate of total pregnancy loss among conception (16.9% vs. 30.3%, *P*=0.003) and a lower rate of biochemical miscarriage (4.8% vs. 12.3%, *P*=0.036) compared with the HRT regimen group. Stratified analyses of reproductive outcomes by ovarian stimulation protocols are showed in **Supplemental Table 1**. For patients who underwent agonist protocol, the letrozole regimen group was associated with higher rates of livebirth (61.9% vs. 49.1%, *P*=0.042) and ongoing pregnancy (63.9% vs. 50.7%, *P*=0.033) than the HRT regimen group, the hMG regimen group had a tendency of increased livebirth rate (56.8% vs. 49.1%) compared with the HRT regimen group. For patients who underwent antagonist protocol, the HRT regimen group also had the lowest rate of livebirth (50.6%, 59.4%, and 55.3%, in HRT group, hMG group, and letrozole group, respectively), although no significant difference.

**Table 2:** Reproductive and neonatal outcomes after FET according to the type of endometrial preparation treatments.

Characteristic	HRT regimen (n = 3540)	hMG regimen (n = 226)	Letrozole regimen (n = 175)	*P* Value
Livebirth (%)	1756 (49.6%)^a^	132 (58.4%)	103 (58.9%)	0.003
Singleton livebirth (%)	1737 (49.1%)	126 (55.8%)	99 (56.6%)	0.028
Twin livebirth (%)	19 (0.5%)^a,b^	6 (2.7%)	4 (2.3%)	0.001
Sex of neonates				0.767
Male (%)	979/1775 (55.2%)	71/138 (51.4%)	64/107 (59.8%)	
Female (%)	796/1775 (44.8%)	67/126 (48.6%)	43/107 (40.2%)	
Birthweight (g)*	3500.0 (3200.0-3800.0)	3475.0 (3100.0-3780.0)	3350.0 (3050.0-3750.0)	0.309
Biochemical pregnancy (%)	2521 (71.2%)	171 (75.7%)	124 (70.9%)	0.351
Clinical pregnancy (%)	2195 (62.0%)	156 (69.0%)	117 (66.9%)	0.053
Ongoing pregnancy (%)	1822 (51.5%)^a^	136 (60.2%)	105 (60.0%)	0.005
Total pregnancy loss among conception (%)	765/2521 (30.3%)^b^	39/171 (22.8%)	21/124 (16.9%)	0.001
Biochemical miscarriage (%)	310/2521 (12.3%)^b^	13/171 (7.6%)	6/124 (4.8%)	0.010
Clinical miscarriage (%)	431/2195 (19.6%)	24/156 (15.4%)	13/117 (11.1%)	0.036
First trimester miscarriage (%)	373/2195 (17.0%)	20/156 (12.8%)	12/117 (10.3%)	0.073
Second trimester miscarriage (%)	58/2195 (2.6%)	4/156 (2.6%)	1/117 (0.9%)	0.602
Ectopic pregnancy (%)	16/2521 (0.6%)	2/171 (1.2%)	1/124 (0.8%)	0.371
Induced delivery (%)	8/2195 (0.4%)	0	1/117 (0.9%)	0.429

HRT, hormone replacement therapy; hMG, human menopausal gonadotropin. ^a^There were significant differences between the HRT group and the hMG group. ^b^There were significant differences between the HRT group and the Letrozole group. *Birthweight of 3 newborn babies in the HRT group and 2 newborn babies in the hMG group was missing.

The comparison of maternal and neonatal complications between the three groups is presented in **Table 3**. No significant difference in maternal and neonatal complications (including preterm delivery, GDM, HDP, SGA, LGA, low birth weight, macrosomia, and congenital anomalies) was observed among the three groups. There were still no significant differences in these complications among the three groups after stratified analyses by ovarian stimulation protocols (**Supplemental Table 2**).

**Table 3:** Maternal and neonatal complications.

Characteristic	HRT regimen (n = 3540)	hMG regimen (n = 226)	Letrozole regimen (n = 175)	*P*-Value
Maternal complications				
Preterm delivery (%)	163/2195 (7.4%)	14/156 (9.0%)	10/117 (8.5%)	0.718
GDM (%)	132/2195 (6.0%)	14/156 (9.0%)	7/117 (6.0%)	0.332
HDP (%)	145/2195 (6.6%)	5/156 (3.2%)	9/117 (7.7%)	0.211
Neonatal complications				
SGA (%)*	47/1772 (2.7%)	4/136 (2.9%)	7/107 (6.5%)	0.065
LGA (%)*	469/1772 (26.5%)	31/136 (22.8%)	23/107 (21.5%)	0.355
Low birth weight (%)*	96/1772 (5.4%)	8/136 (5.9%)	7/107 (6.5%)	0.868
Macrosomia (%)*	228/1772 (12.9%)	16/136 (11.8%)	14/107 (13.1%)	0.929
Congenital anomalies (%)	20/1775 (1.1%)	4/138 (2.9%)	1/107(0.9%)	0.185

HRT, hormone replacement therapy; hMG, human menopausal gonadotropin; GDM, gestational diabetes mellitus; HDP, hypertensive disorders of pregnancy; SGA, small-for-gestation-age; LGA, large-for-gestation-age. *Birthweight of 3 newborn babies in the HRT group and 2 newborn babies in the hMG group was missing.

After adjustment for the above-mentioned confounding factors (**Table 4**), the hMG regimen group [adjusted odds ratio (aOR): 1.43; 95% confidence interval (CI): 1.09-1.89)] and the letrozole regimen group (aOR: 1.42; 95% CI: 1.04-1.93) had a higher rate of livebirth, compared with the group with HRT regimen. The rate of ongoing pregnancy was also higher in the hMG regimen group (aOR: 1.44; 95% CI: 1.09-1.91) and the letrozole regimen group (aOR: 1.38; 95% CI: 1.01-1.89) than the HRT group. Both the hMG regimen group (aOR: 0.69; 95% CI: 0.48-1.00) and the letrozole regimen group (aOR: 0.48; 95% CI: 0.30-0.78) were associated with a lower risk of pregnancy loss. The risks of biochemical miscarriage (aOR: 0.39; 95% CI: 0.17-0.89) and clinical miscarriage (aOR: 0.50; 95% CI: 0.28-0.90) were reduced in letrozole regimen group in comparison to the HRT regimen group. Stratified logistic regression of pregnancy outcomes by ovarian stimulation protocols is showed in **Supplemental Table 3**. For patients who underwent agonist protocol, the letrozole regimen group also had higher rates of livebirth rate (aOR: 1.70; 95% CI: 1.11-2.59), clinical pregnancy (aOR: 1.71; 95% CI: 1.08-2.71), and ongoing pregnancy (aOR: 1.76; 95% CI: 1.15-2.72), and a lower rate of total pregnancy loss among conception (aOR: 0.54; 95% CI: 0.30-0.97) compared with the HRT regimen group. For patients who underwent antagonist protocol, the letrozole regimen group was associated with a lower rate of total pregnancy loss among conception (aOR: 0.35; 95% CI: 0.15-0.83) compared with the HRT regimen group.

**Table 4:** Logistic regression of pregnancy outcomes.

Characteristic	hMG regimen vs. HRT regimen		Letrozole regimen vs. HRT regimen	
	*aOR* (95% CI)	*P* Value	*aOR* (95% CI)	*P* Value
Livebirth	1.43 (1.09-1.89)	0.011	1.42 (1.04-1.93)	0.029
Singleton livebirth	1.30 (0.99-1.71)	0.059	1.31 (0.97-1.79)	0.083
Twin livebirth	5.29 (2.06-13.58)	0.001	5.40 (1.77-16.53)	0.003
Biochemical pregnancy	1.31 (0.95-1.79)	0.099	0.99 (0.71-1.38)	0.947
Clinical pregnancy	1.39 (1.04-1.86)	0.029	1.21 (0.87-1.67)	0.252
Ongoing pregnancy	1.44 (1.09-1.91)	0.010	1.38 (1.01-1.89)	0.042
Total pregnancy loss among conception	0.69 (0.48-1.00)	0.048	0.48 (0.30-0.78)	0.003
Biochemical miscarriage	0.58 (0.37-1.04)	0.069	0.39 (0.17-0.89)	0.025
Clinical pregnancy loss	0.76 (0.49-1.20)	0.234	0.50 (0.28-0.90)	0.020

aOR, adjusted odds ratio; CI, confidence interval. Analyses were adjusted for age, body mass index, duration of infertility, gravidity, year of treatment, and number of oocytes retrieved.

## Discussion

In this study, we found that both the hMG regimen group and the letrozole regimen group were associated with a higher rate of livebirth and a lower rate of pregnancy loss compared with the group with HRT regimen. When stratified by ovarian stimulation protocols, the letrozole regimen was associated with a higher rate of livebirth rate in patients who underwent agonist protocol, and a lower rate of pregnancy loss in patients who underwent antagonist protocol, as compared with the HRT regimen group. We did not observe significant difference in the incidences of maternal and neonatal complications among these three groups. Thus, ovulation induction regimen may be a better choice for endometrial preparation for frozen single-blastocyst transfer in women with PCOS.

Our results were consistent with several previous studies conducted in patients with ovulation disorders (13, 15, 30, 31). Man et al. reported a higher rate of live birth in hMG regimen compared with HRT regimen in patients with PCOS who undergo frozen single-blastocyst transfer (13). The study by Hu et al. showed that that in 120 patients with PCOS, letrozole ovulation induction regimen was associated with higher rates of implantation, clinical pregnancy and ongoing pregnancy, when compared with HRT regimen (30). Zhang and coworkers observed that in patients with PCOS, most of whom underwent cleavage stage embryo transfer, the letrozole regimen group was associated with a higher rate of livebirth and a lower rate of pregnancy loss than the HRT regimen group (15). The study by Li et al. also demonstrated higher implantation rate, clinical pregnancy rate, and livebirth rate but a lower abortion rate with letrozole ovulation induction regimen cycles compared with HRT cycles in patients with ovulation disorders (31).

During HRT cycles, high doses of exogenous estrogen and progesterone are needed for luteal phase support in the first trimester. Although it has been proposed that the use of HRT regimen should be abandoned in ovulatory women (32), HRT regimen still is the first choice for women who have irregular menstrual cycles. Our study provides additional evidence for the previous findings that the HRT regimen was associated with a lower rate of livebirth and a higher rate of pregnancy loss compared with the ovulation induction regimens for women with PCOS, even after single-blastocyst transfer. The underlying mechanism is unclear. However, the absence of a corpus luteum may plays a role. Besides production of steroid hormone, corpus luteum has potential influence on angiogenesis and immunology (33). The study by Conrad et al. discovered that the absence of a corpus luteum in IVF cycles was related to maternal cardiovascular dysregulation in early age of pregnancy (34). The suboptimal maternal cardiovascular function may contribute to compromised pregnancy outcomes. In addition, the possibility that inadequate or excessive dosage or imprecise timing of estrogen and progesterone administration in HRT cycles may also adversely affect endometrial function and subsequently lead to adverse pregnancy outcomes (35). It was demonstrated that estrogen administration exceeded 28 days or high serum estradiol levels (≥400 pg/ml) prior to progesterone administration was associated with a decreased live birth rate during frozen blastocyst transfer cycles (36, 37).

Although accumulated evidences showed HRT regimen for endometrial preparation was associated with an increased risk of HDP compared with natural regimen where the corpus luteum exists in ovulatory women (7), there was no difference in the risk of HDP in our study when the corpus luteum status varied. Women with PCOS are at higher risk of adverse pregnancy and birth outcomes (38), in that condition, the negative effects caused by lack of corpus luteum may be aggravated, the establishment and maintaining of pregnancy was compromised, and subsequently lead to increased pregnancy loss and decreased incidence for acquiring livebirth, while the difference of the risk of HDP became unsignificant. The study by Zhang et al. showed that HRT regimen for endometrial preparation was associated with an increased risk of HDP compared with ovulation regimen among women with PCOS (16). However, most patients in their study underwent double cleavage stage embryos transfer, and above one third patients achieved twin delivery.

In ovulation regimens, the receptivity of endometrium to embryo implantation relies on endogenous estrogen, progesterone, and other factors produced during the maturity of a dominant follicle, ovulation, and subsequent formulation of a corpus luteum. Accordant with the recent meta-analysis (39), our study showed that both hMG regimen and letrozole regimen result in a higher livebirth rate compared with HRT regimen, while hMG regimen and letrozole regimen achieved comparable rate of livebirth. However, when stratified by ovarian stimulation protocols, the difference in terms of livebirth between hMG regimen and HRT regimen was attenuated. Up to now, which medicine is superior for ovulation induction has yet to be determined. However, infertile people with PCOS might benefit from the use of letrozole (40).

Our study indicated the endometrial thickness before embryo transfer was thicker in the letrozole group compared with the HRT group. Letrozole may induce a molecular response in the endometrium of PCOS patients in the Wnt/B-catenin pathway, which plays a pivotal role in cell proliferation, differentiation, motility, survival, embryo implantation, and placental development (41, 42). Secondly, letrozole can decrease the production of estrogen, as a result, intraovarian and serum estrogen levels were reduced (43). Low estrogen levels left a large number of estrogen receptors unoccupied, in order to increase sensitivity to subsequent estrogen rise (44). Furthermore, letrozole have a positive effect on the expression of molecules related to endometrial receptivity (19). Promoted integrin expression and improved pregnancy and implantation rates was founded after co-treatment with letrozole in patient with defected endometrial receptivity (45). Thus, the treatment with letrozole for endometrial preparation in patients with PCOS may offer greater potential.

A strength of our study was its large sample size and data integrity. Additionally, we only included patients who underwent frozen single-blastocyst transfer, which has been increasingly advocated. We acknowledge that this study has limitations. First, it was a retrospective observational study, although we only enrolled women who underwent their first FET cycles at the first attempts of IVF, and adjusted confounders with multivariable logistic regression, the bias and confounding factors may still influence the results of our study. Furthermore, due to the great sample difference among the three groups, we were at risks for both a Type 1 and a Type 2 error. Additionally, as a result of small sample, when it comes to maternal and neonatal complications, there may lack of statistics validity. Large sample and multicenter randomized controlled trial are needed to assess the efficacy of ovulation induction regimen as a method of endometrial preparation for FET in patients with PCOS.

## Conclusion

We found that for patients with PCOS who underwent frozen single-blastocyst transfer, both hMG ovulation induction regimen and letrozole ovulation induction regimen for endometrial preparation were associated with better pregnancy outcomes compared with HRT regimen, such as a higher livebirth rate and a lower pregnancy loss rate. Further studies are warranted to explore which ovulation induction regimen is superior for endometrial preparation before FET.

## Data availability statement

The original contributions presented in the study are included in the article/**Supplementary Material**. Further inquiries can be directed to the corresponding author.

## Ethics statement

The studies involving human participants were reviewed and approved by The Institutional Ethics Committee of the Center for Reproductive Medicine of Shandong University. The patients/participants provided their written informed consent to participate in this study. Written informed consent was obtained from the individual(s) for the publication of any potentially identifiable images or data included in this article.

## Author contributions

DW supervised the entire study and revised the manuscript; YN collected data, analyzed the data, and drafted the manuscript; DZ, YW, LS, and JZ collected data and analyzed the data. All authors have been involved in interpreting the data and have approved the final version.

## Funding

This work was supported by grants from the National Natural Science Foundation of China (82071718).

## Acknowledgments

The authors thank the patients and staff of the Center for Reproductive Medicine of Shandong University for their cooperation and support.

## Conflict of interest

The authors declare that the research was conducted in the absence of any commercial or financial relationships that could be construed as a potential conflict of interest.

## Publisher’s note

All claims expressed in this article are solely those of the authors and do not necessarily represent those of their affiliated organizations, or those of the publisher, the editors and the reviewers. Any product that may be evaluated in this article, or claim that may be made by its manufacturer, is not guaranteed or endorsed by the publisher.

## References

[1] Lizneva D Suturina L Walker W Brakta S Gavrilova-Jordan L Azziz R. Criteria, prevalence, and phenotypes of polycystic ovary syndrome. Fertil Steril (2016) 106(1):6–15. doi: 10.1016/j.fertnstert.2016.05.003 27233760

[2] Chen ZJ Shi Y Sun Y Zhang B Liang X Cao Y. Fresh versus frozen embryos for infertility in the polycystic ovary syndrome. N Engl J Med (2016) 375(6):523–33. doi: 10.1056/NEJMoa1513873 27509101

[3] Lin J Guo H Wang B Chen Q Zhu Q. Neonatal outcomes in women with polycystic ovary syndrome after frozen-thawed embryo transfer. Fertil Steril (2021) 115(2):447–54. doi: 10.1016/j.fertnstert.2020.08.1435 33272629

[4] Wang N Zhao X Ma M Zhu Q Wang Y. Effect of day 3 and day 5/6 embryo quality on the reproductive outcomes in the single vitrified embryo transfer cycles. Front Endocrinol (Lausanne) (2021) 12:641623. doi: 10.3389/fendo.2021.641623 34046010 PMC8147686

[5] Groenewoud ER Cantineau AE Kollen BJ Macklon NS Cohlen BJ. What is the optimal means of preparing the endometrium in frozen-thawed embryo transfer cycles? A systematic review and meta-analysis. Hum Reprod Update (2013) 19(5):458–70. doi: 10.1093/humupd/dmt030 23820515

[6] Dal Prato L Borini A Cattoli M Bonu MA Sciajno R Flamigni C. Endometrial preparation for frozen-thawed embryo transfer with or without pretreatment with gonadotropin-releasing hormone agonist. Fertil Steril (2002) 77(5):956–60. doi: 10.1016/s0015-0282(02)02960-6 12009350

[7] Busnelli A Schirripa I Fedele F Bulfoni A Levi-Setti PE. Obstetric and perinatal outcomes following programmed compared to natural frozen-thawed embryo transfer cycles: A systematic review and meta-analysis. Hum Reprod (2022) 37(7):1619–41. doi: 10.1093/humrep/deac073 35553678

[8] Cerrillo M Herrero L Guillen A Mayoral M Garcia-Velasco JA. Impact of endometrial preparation protocols for frozen embryo transfer on live birth rates. Rambam Maimonides Med J (2017) 8(2). doi: 10.5041/RMMJ.10297 PMC5415366 28467767

[9] Mounce G McVeigh E Turner K Child TJ. Randomized, controlled pilot trial of natural versus hormone replacement therapy cycles in frozen embryo replacement *in vitro* fertilization. Fertil Steril (2015) 104(4):915–20.e1. doi: 10.1016/j.fertnstert.2015.07.1131 26255087

[10] Groenewoud ER Cohlen BJ Al-Oraiby A Brinkhuis EA Broekmans FJ de Bruin JP. A randomized controlled, non-inferiority trial of modified natural versus artificial cycle for cryo-thawed embryo transfer. Hum Reprod (2016) 31(7):1483–92. doi: 10.1093/humrep/dew120 PMC5853593 27179265

[11] Aslih N Dorzia D Atzmon Y Estrada D Ellenbogen A Bilgory A. Ovulatory-based fet cycles may achieve higher pregnancy rates in the general population and among anovulatory women. J Clin Med (2021) 10(4). doi: 10.3390/jcm10040703 PMC7916855 33670133

[12] Hosseini-Najarkolaei A Moini A Kashani L Farid Mojtahedi M Hosseini-Najarkolaee E Salehi E. The effect of letrozole versus artificial hormonal endometrial preparation on pregnancy outcome after frozen-thawed embryos transfer cycles: A randomized clinical trial. Reprod Biol Endocrinol (2020) 18(1):115. doi: 10.1186/s12958-020-00675-z 33218350 PMC7678072

[13] Man Y Bian Y Zhao S Zhao R Xu X Wei D. The effect of different endometrial preparations on women with polycystic ovary syndrome undergoing initial frozen embryo transfer: A historical cohort analysis. Acta Obstet Gynecol Scand (2021) 100(6):1116–23. doi: 10.1111/aogs.14058 33616957

[14] Li L Gao DD Zhang Y Song JY Sun ZG. Comparison of stimulated cycles with low dose r-fsh versus hormone replacement cycles for endometrial preparation prior to frozen-thawed embryo transfer in young women with polycystic ovarian syndrome: A single-center retrospective cohort study from China. Drug Des Devel Ther (2021) 15:2805–13. doi: 10.2147/dddt.S317545 PMC8253980 34234412

[15] Zhang J Liu H Wang Y Mao X Chen Q Fan Y. Letrozole use during frozen embryo transfer cycles in women with polycystic ovary syndrome. Fertil Steril (2019) 112(2):371–7. doi: 10.1016/j.fertnstert.2019.04.014 31126712

[16] Zhang J Wei M Bian X Wu L Zhang S Mao X. Letrozole-induced frozen embryo transfer cycles are associated with a lower risk of hypertensive disorders of pregnancy among women with polycystic ovary syndrome. Am J Obstet Gynecol (2021) 225(1):59.e1–.e9. doi: 10.1016/j.ajog.2021.01.024 33529574

[17] Franik S Eltrop SM Kremer JA Kiesel L Farquhar C. Aromatase inhibitors (Letrozole) for subfertile women with polycystic ovary syndrome. Cochrane Database Syst Rev (2018) 5:CD010287. doi: 10.1002/14651858.CD010287.pub3 29797697 PMC6494577

[18] Weiss NS Kostova E Nahuis M Mol BWJ van der Veen F van Wely M. Gonadotrophins for ovulation induction in women with polycystic ovary syndrome. Cochrane Database Syst Rev (2019) 1(1):Cd010290. doi: 10.1002/14651858.CD010290.pub3 30648738 PMC6353048

[19] Lin J Wang N Huang J Cai R Fan Y Kuang Y. Pregnancy and neonatal outcomes of hmg stimulation with or without letrozole in endometrial preparation for frozen-thawed embryo transfer in ovulatory women: A Large retrospective cohort study. Drug Des Devel Ther (2019) 13:3867–77. doi: 10.2147/dddt.S212235 PMC6861551 31814708

[20] Wallace KL Johnson V Sopelak V Hines R. Clomiphene citrate versus letrozole: Molecular analysis of the endometrium in women with polycystic ovary syndrome. Fertil Steril (2011) 96(4):1051–6. doi: 10.1016/j.fertnstert.2011.07.1092 21851939

[21] Al-Obaidi MT Ali ZH Al-Saadi WI Al-Wasiti EAR Al-Aubaidy H. Impact of letrozole versus clomiphene citrate on endometrial receptivity in Iraqi women with polycystic ovarian syndrome. J Clin Pharm Ther (2019) 44(4):618–22. doi: 10.1111/jcpt.12831 30868612

[22] Wang L Wen X Lv S Zhao J Yang T Yang X. Comparison of endometrial receptivity of clomiphene citrate versus letrozole in women with polycystic ovary syndrome: A randomized controlled study. Gynecol Endocrinol (2019) 35(10):862–5. doi: 10.1080/09513590.2019.1612358 31081404

[23] Rotterdam EA-SPcwg. Revised 2003 consensus on diagnostic criteria and long-term health risks related to polycystic ovary syndrome (Pcos). Hum Reprod (2004) 19(1):41–7. doi: 10.1093/humrep/deh098 14688154

[24] Qin Y Zhao Z Sun M Geng L Che L Chen ZJ. Association of basal serum testosterone levels with ovarian response and *in vitro* fertilization outcome. Reprod Biol Endocrinol (2011) 9:9. doi: 10.1186/1477-7827-9-9 21247501 PMC3031218

[25] Zhang B Meng Y Jiang X Liu C Zhang H Cui L. Ivf outcomes of women with discrepancies between age and serum anti-müllerian hormone levels. Reprod Biol Endocrinol (2019) 17(1):58. doi: 10.1186/s12958-019-0498-3 31311571 PMC6636016

[26] Puissant F Van Rysselberge M Barlow P Deweze J Leroy F. Embryo scoring as a prognostic tool in ivf treatment. Hum Reprod (1987) 2(8):705–8. doi: 10.1093/oxfordjournals.humrep.a136618 3437050

[27] Gardner DK Lane M Schoolcraft WB. Physiology and culture of the human blastocyst. J Reprod Immunol (2002) 55(1-2):85–100. doi: 10.1016/s0165-0378(01)00136-x 12062824

[28] Metzger BE Gabbe SG Persson B Buchanan TA Catalano PA Damm P. International association of diabetes and pregnancy study groups recommendations on the diagnosis and classification of hyperglycemia in pregnancy. Diabetes Care (2010) 33(3):676–82. doi: 10.2337/dc09-1848 PMC2827530 20190296

[29] Garovic VD Dechend R Easterling T Karumanchi SA McMurtry Baird S Magee LA. Hypertension in pregnancy: Diagnosis, blood pressure goals, and pharmacotherapy: A scientific statement from the American heart association. Hypertension (2022) 79(2):e21–41. doi: 10.1161/hyp.0000000000000208 PMC9031058 34905954

[30] Hu YJ Chen YZ Zhu YM Huang HF. Letrozole stimulation in endometrial preparation for cryopreserved-thawed embryo transfer in women with polycystic ovarian syndrome: A pilot study. Clin Endocrinol (Oxf) (2014) 80(2):283–9. doi: 10.1111/cen.12280 23808904

[31] Li SJ Zhang YJ Chai XS Nie MF Zhou YY Chen JL. Letrozole ovulation induction: An effective option in endometrial preparation for frozen-thawed embryo transfer. Arch Gynecol Obstet (2014) 289(3):687–93. doi: 10.1007/s00404-013-3044-0 24121690

[32] von Versen-Höynck F Griesinger G. Should any use of artificial cycle regimen for frozen-thawed embryo transfer in women capable of ovulation be abandoned: Yes, but what’s next for fet cycle practice and research? Hum Reprod (2022) 37(8):1697–703. doi: 10.1093/humrep/deac125 35640158

[33] Conrad KP Graham GM Chi YY Zhai X Li M Williams RS. Potential influence of the corpus luteum on circulating reproductive and volume regulatory hormones, angiogenic and immunoregulatory factors in pregnant women. Am J Physiol Endocrinol Metab (2019) 317(4):E677–E85. doi: 10.1152/ajpendo.00225.2019 PMC6842916 31408378

[34] Conrad KP Petersen JW Chi YY Zhai X Li M Chiu KH. Maternal cardiovascular dysregulation during early pregnancy after *in vitro* fertilization cycles in the absence of a corpus luteum. Hypertension (2019) 74(3):705–15. doi: 10.1161/HYPERTENSIONAHA.119.13015 PMC6687559 31352818

[35] Conrad KP von Versen-Höynck F Baker VL. Potential role of the corpus luteum in maternal cardiovascular adaptation to pregnancy and preeclampsia risk. Am J Obstet Gynecol (2022) 226(5):683–99. doi: 10.1016/j.ajog.2021.08.018 34437863

[36] Zhou R Zhang X Huang L Zhu X Dong M Liu W. Association between serum estradiol levels prior to progesterone administration in artificial frozen-thawed blastocyst transfer cycles and live birth rate: A retrospective study. Bjog (2021) 128(13):2092–100. doi: 10.1111/1471-0528.16777 34047447

[37] Bourdon M Santulli P Kefelian F Vienet-Legue L Maignien C Pocate-Cheriet K. Prolonged estrogen (E2) treatment prior to frozen-blastocyst transfer decreases the live birth rate. Hum Reprod (2018) 33(5):905–13. doi: 10.1093/humrep/dey041 29529202

[38] Roos N Kieler H Sahlin L Ekman-Ordeberg G Falconer H Stephansson O. Risk of adverse pregnancy outcomes in women with polycystic ovary syndrome: Population based cohort study. Bmj (2011) 343:d6309. doi: 10.1136/bmj.d6309 21998337 PMC3192872

[39] Zhang Y Wu L Li TC Wang CC Zhang T Chung JPW. Systematic review update and meta-analysis of randomized and non-randomized controlled trials of ovarian stimulation versus artificial cycle for endometrial preparation prior to frozen embryo transfer in women with polycystic ovary syndrome. Reprod Biol Endocrinol (2022) 20(1):62. doi: 10.1186/s12958-022-00931-4 35366912 PMC8976372

[40] Palomba S. Aromatase inhibitors for ovulation induction. J Clin Endocrinol Metab (2015) 100(5):1742–7. doi: 10.1210/jc.2014-4235 25710566

[41] Mehdinejadiani S Amidi F Mehdizadeh M Barati M Pazhohan A Alyasin A. Effects of letrozole and clomiphene citrate on wnt signaling pathway in endometrium of polycystic ovarian syndrome and healthy womendagger. Biol Reprod (2019) 100(3):641–8. doi: 10.1093/biolre/ioy187 30184105

[42] Mehdinejadiani S Amidi F Mehdizadeh M Barati M Safdarian L Aflatoonian R. The effects of letrozole and clomiphene citrate on ligands expression of Wnt3, Wnt7a, and Wnt8b in proliferative endometrium of women with polycystic ovarian syndrome. Gynecol Endocrinol (2018) 34(9):775–80. doi: 10.1080/09513590.2018.1446934 29510649

[43] Garcia-Velasco JA. The use of aromatase inhibitors in in vitro fertilization. Fertil Steril (2012) 98(6):1356–8. doi: 10.1016/j.fertnstert.2012.09.042 23062732

[44] Tatsumi T Jwa SC Kuwahara A Irahara M Kubota T Saito H. Pregnancy and neonatal outcomes following letrozole use in frozen-thawed single embryo transfer cycles. Hum Reprod (2017) 32(6):1244–8. doi: 10.1093/humrep/dex066 28398491

[45] Miller PB Parnell BA Bushnell G Tallman N Forstein DA Higdon HL 3rd. Endometrial receptivity defects during ivf cycles with and without letrozole. Hum Reprod (2012) 27(3):881–8. doi: 10.1093/humrep/der452 PMC3279128 22246449

