# Supplementary material for: Ovulation induction regimens are associated with a higher rate of livebirth after frozen single-blastocyst transfer among women with polycystic ovary syndrome
Source: Front Endocrinol (Lausanne). 2022 Aug 15;13:987813. doi: 10.3389/fendo.2022.987813 (PMC9423737; doi:10.3389/fendo.2022.987813)
Supplement: Supplementary file 1 [file Table_1.docx]

Supplemental Table 1 Stratified analyses of reproductive outcomes by ovarian stimulation protocols.

| Characteristic | Agonist protocol | | | | Antagonist protocol | | | |
| --- | --- | --- | --- | --- | --- | --- | --- | --- |
|  | HRT regimen  (n = 1893) | hMG regimen  (n = 118) | Letrozole regimen  (n = 97) | *P* Value | HRT regimen  (n = 1610) | hMG regimen  (n = 106) | Letrozole regimen  (n = 76) | *P* Value |
| Livebirth (%) | 930 (49.1%) ^b^ | 67 (56.8%) | 60 (61.9%) | 0.017 | 815 (50.6%) | 63 (59.4%) | 42 (55.3%) | 0.167 |
| Singleton livebirth (%) | 918 (48.5%) | 65 (55.1%) | 56 (57.7%) | 0.089 | 808 (50.2%) | 59 (55.7%) | 42 (55.3%) | 0.397 |
| Twin livebirth (%) | 12 (0.6%) ^b^ | 2 (1.7%) | 4 (4.1%) | <0.001 | 7 (0.4%) ^a^ | 4 (3.8%) | 0 (0.0%) | 0.005 |
| Sex of neonates |  |  |  | 0.864 |  |  |  | 0.299 |
| Male (%) | 515/918 (56.1%) | 38/65 (58.5%) | 33/56 (58.9%) |  | 440/808 (54.5%) | 26/59 (44.1%) | 22/42 (52.4%) |  |
| Female (%) | 403/918 (43.9%) | 27/65 (41.5%) | 23/56 (41.1%) |  | 368/808 (45.5%) | 33/59 (55.9%) | 20/42 (47.6%) |  |
| Birthweight (g) | 3500.0 (3150.0-3800.0) | 3450.0 (3100.0-3790.0) | 3410.0 (3100.0-3800.0) | 0.489 | 3500.0 (3180.0-3800.0) | 3400.0 (3100.0-3780.0) | 3500.0 (3200.0-3800.0) | 0.133 |
| Biochemical pregnancy (%) | 1340 (70.8%) | 88 (74.6%) | 75 (77.3%) | 0.275 | 1158 (71.9%) | 81 (76.4%) | 48 (63.2%) | 0.140 |
| Clinical pregnancy (%) | 1166 (61.6%) | 80 (67.8%) | 71 (73.2%) | 0.403 | 1010 (62.7%) | 74 (69.8%) | 45 (59.2%) | 0.269 |
| Ongoing pregnancy (%) | 960 (50.7%) ^b^ | 68 (57.6%) | 62 (63.9%) | 0.017 | 849 (52.7%) | 66 (62.3%) | 42 (55.3%) | 0.154 |
| Total pregnancy loss among conception (%) | 410/1340 (30.6%) | 21/88 (23.9%) | 15/75 (20.0%) | 0.069 | 343/1158 (29.6%) ^b^ | 18/81 (22.2%) | 6/48 (12.5%) | 0.016 |
| Biochemical miscarriage (%) | 164/1340 (12.2%) | 6/88 (6.8%) | 4/75 (5.3%) | 0.068 | 142/1158 (12.3%) | 7/81 (8.6%) | 2/48 (4.2%) | 0.156 |
| Clinical miscarriage (%) | 230/1166 (19.7%) | 13/80 (16.3%) | 10/71 (14.1%) | 0.396 | 193/1010 (19.1%) | 11/74 (14.9%) | 3/45 (6.7%) | 0.078 |
| First trimester miscarriage (%) | 206/1166 (17.7%) | 12/80 (15.0%) | 9/71 (12.7%) | 0.480 | 161/1010 (15.9%) | 8/74 (10.8%) | 3/45 (6.7%) | 0.131 |
| Second trimester miscarriage (%) | 24/1166 (2.1%) | 1/80 (1.3%) | 1/71 (1.4%) | >0.999 | 32/1010 (3.2%) | 3/74 (4.1%) | 0/45 (0.0%) | 0.501 |
| Ectopic pregnancy (%) | 10/1340 (0.7%) | 2/88 (2.35%) | 0/75 (0.0%) | 0.256 | 6/1158 (0.5%) | 0/81 (0.0%) | 1/48 (2.1%) | 0.289 |
| Induced delivery (%) | 6/1166 (0.5%) | 0/80 (0.0%) | 1/71 (1.4%) | 0.369 | 2/1010 (0.2%) | 0/74 (0.0%) | 0/45 (0.0%) | >0.999 |

Note: HRT, hormone replacement therapy; hMG, human menopausal gonadotropin.

^a^ There were significant differences between the HRT group and the hMG group.

^b^ There were significant differences between the HRT group and the Letrozole group.

Supplemental Table 2 Stratified analyses of maternal and neonatal complications by ovarian stimulation protocols.

| Characteristic | Agonist protocol | | | | Antagonist protocol | | | |
| --- | --- | --- | --- | --- | --- | --- | --- | --- |
|  | HRT regimen  (n = 1893) | hMG regimen  (n = 118) | Letrozole regimen  (n = 97) | *P*-Value | HRT regimen  (n = 1610) | hMG regimen  (n = 106) | Letrozole regimen  (n = 76) | *P*-Value |
| Maternal complications |  |  |  |  |  |  |  |  |
| Preterm delivery (%) | 68/1166 (5.8%) | 7/80 (8.8%) | 5/71 (7.0%) | 0.435 | 95/1010 (9.4%) | 7/74 (9.5%) | 5/45 (11.1%) | 0.846 |
| GDM (%) | 65/1166 (5.6%) | 7/80 (8.8%) | 2/71 (2.8%) | 0.301 | 67/1010 (6.6%) | 7/74 (9.5%) | 5/45 (11.1%) | 0.252 |
| HDP (%) | 72/1166 (6.2%) | 3/80 (3.8%) | 4/71 (5.6%) | 0.787 | 72/1010 (7.1%) | 2/74 (2.7%) | 5/45 (11.1%) | 0.182 |
| Neonatal complications |  |  |  |  |  |  |  |  |
| SGA (%) | 32/941 (3.4%) | 2/68 (2.9%) | 5/64 (7.8%) | 0.162 | 15/820 (1.8%) | 1/66 (1.5%) | 2/42 (4.8%) | 0.304 |
| LGA (%) | 256/941 (27.2%) | 14/68 (20.6%) | 16/64 (25.0%) | 0.500 | 210/820 (25.6%) | 16/66 (24.2%) | 7/42 (16.7%) | 0.465 |
| Low birth weight (%) | 42/941 (3.1%) | 2/68 (2.9%) | 5/64 (7.8%) | 0.335 | 54/820 (6.6%) | 6/66 (9.1%) | 2/42 (4.8%) | 0.696 |
| Macrosomia (%) | 117/941 (12.4%) | 8/68 (11.8%) | 10/64 (15.6%) | 0.721 | 109/820 (13.3%) | 8/66 (12.1%) | 4/42 (9.5%) | 0.869 |
| Congenital anomalies (%) | 9/942 (1.0%) | 0 | 0 | >0.999 | 11/822 (1.3%) ^a^ | 4/67 (6.0%) | 1/42 (2.4%) | 0.024 |

Note: HRT, hormone replacement therapy; hMG, human menopausal gonadotropin; GDM, gestational diabetes mellitus; HDP, hypertensive disorders of pregnancy; SGA, small-for-gestation-age; LGA, large-for-gestation-age.

^a^ There were significant differences between the HRT group and the hMG group.

Supplemental Table 3 Stratified logistic regression of pregnancy outcomes by ovarian stimulation protocols.

| Characteristic | Agonist protocol | | | | Antagonist protocol | | | |
| --- | --- | --- | --- | --- | --- | --- | --- | --- |
|  | hMG regimen *vs.* HRT regimen | | Letrozole regimen *vs.* HRT regimen | | hMG regimen *vs.* HRT regimen | | Letrozole regimen *vs.* HRT regimen | |
|  | *aOR* (95% CI) | *P* Value | *aOR* (95% CI) | *P* Value | *aOR* (95% CI) | *P* Value | *aOR* (95% CI) | *P* Value |
| Livebirth | 1.39 (0.95-2.04) | 0.090 | 1.70 (1.11-2.59) | 0.015 | 1.37 (0.92-2.06) | 0.125 | 1.18 (0.74-1.88) | 0.487 |
| Singleton livebirth | 1.37 (0.94-2.01) | 0.105 | 1.48 (0.97-2.26) | 0.067 | 1.19 (0.80-1.77) | 0.401 | 1.20 (0.75-1.92) | 0.438 |
| Twin livebirth | 2.97 (0.64-13.87) | 0.166 | 9.58 (2.75-33.39) | <0.001 | 10.55 (2.97-37.46) | <0.001 | 0 | 0.997 |
| Biochemical pregnancy | 1.31 (0.85-2.03) | 0.224 | 1.51 (0.92-2.46) | 0.103 | 1.24 (0.78-1.97) | 0.369 | 0.66 (0.41-1.07) | 0.088 |
| Clinical pregnancy | 1.36 (0.91-2.04) | 0.134 | 1.71 (1.08-2.71) | 0.023 | 1.34 (0.87-2.06) | 0.182 | 0.84 (0.53-1.35) | 0.479 |
| Ongoing pregnancy | 1.38 (0.94-2.03) | 0.099 | 1.76 (1.15-2.72) | 0.010 | 1.45 (0.96-2.18) | 0.076 | 1.08 (0.68-1.72) | 0.740 |
| Total pregnancy loss among conception | 0.73 (0.44-1.21) | 0.217 | 0.54 (0.30-0.97) | 0.038 | 0.71 (0.41-1.22) | 0.218 | 0.35 (0.15-0.83) | 0.018 |
| Biochemical miscarriage | 0.53 (0.23-1.24) | 0.143 | 0.39 (0.14-1.09) | 0.072 | 0.68 (0.31-1.52) | 0.351 | 0.33 (0.68-0.31) | 0.126 |
| Clinical pregnancy loss | 0.80 (0.43-1.48) | 0.471 | 0.63 (0.31-1.26) | 0.189 | 0.78 (0.40-1.51) | 0.452 | 0.30 (0.09-1.00) | 0.049 |

Note: *aOR*, adjusted odds ratio; CI, confidence interval.

Analyses were adjusted for age, body mass index, duration of infertility, gravidity, year of treatment, and number of oocytes retrieved.
